# The Dynamic Transmission of Simbu Group Viruses in New South Wales, Australia

**DOI:** 10.1155/tbed/9214955

**Published:** 2026-06-24

**Authors:** Tiffany W. O’Connor, Paul M. Hick, Jenny-Ann L. M. L. Toribio, Alison J. Peel, Peter D. Kirkland, Deborah S. Finlaison

**Affiliations:** ^1^ Sydney School of Veterinary Science, The University of Sydney, Camden, New South Wales, Australia, sydney.edu.au; ^2^ NSW Department of Primary Industries and Regional Development, Elizabeth Macarthur Agricultural Institute, Menangle, New South Wales, Australia

**Keywords:** Aino virus, Akabane virus, Douglas virus, Orthobunyavirus, Peaton virus, sentinel surveillance, Shamonda virus, Simbu serogroup, Tinaroo virus

## Abstract

Arthropod‐borne orthobunyaviruses belonging to the Simbu serogroup can cause congenital and neurological disease in livestock. Across Australia, the National Arbovirus Monitoring Program (NAMP) conducts surveillance for veterinary‐important arboviruses. Our study complements this broader surveillance framework by investigating the virus‐specific transmission patterns of Simbu group viruses (SGVs) present in New South Wales (NSW). To describe the distribution of six SGVs: Akabane, Aino, Douglas, Peaton, Tinaroo, and a recently recognised Shamonda‐like virus, we examined 24 years of serological data (2000–2023) from NSW sentinel cattle. The transmission of a Simbu serogroup virus was detected during every monitoring period (October–July), progressing from north to south along the NSW coastline. Inland transmission west of the Great Dividing Range (GDR) was intermittent (2012–2013, 2020, and 2022), when SGV distribution extended across the mountain range or progressed down the western slopes from Queensland. From 2014, repeated seasonal SGV transmission extended to the far south coast of NSW. Peaton virus was the most frequently detected and widely distributed virus. Since testing for the newly detected Shamonda‐like virus started in 2019, this virus has been widely detected across coastal NSW and often in herds also infected with Douglas or Peaton viruses. Discrepancies between the ELISA and virus neutralisation test (VNT) results suggest that this virus could have been present in NSW as early as 2012. From 2017, Akabane virus has a sporadic distribution. The distribution for both Aino and Tinaroo viruses was sporadic (2005–2023). These findings highlight the importance of long‐term, virus‐specific surveillance in identifying emerging variants and detecting exotic incursions.

## 1. Introduction

Among the arboviruses of veterinary interest, orthobunyaviruses in the Simbu serogroup have caused epidemics of congenital malformations with associated reproductive losses and neurological disease in livestock. Akabane virus (*Orthobunyavirus akabaneense*) has caused disease outbreaks in Australia, Asia, and the Middle East [1–4]. Schmallenberg virus (*Orthobunyavirus schmallenbergense*) causes similar outbreaks in ruminant production systems across Europe [5, 6]. Other Simbu group viruses (SGVs), including Aino (*Orthobunyavirus ainoense*), Peaton (*Orthobunyavirus peachesterense*), Shamonda (*O. schmallenbergense*), and Shuni (*Orthobunyavirus shuniense*), can also be pathogenic in cattle, sheep, goats, and horses [7]. Understanding the transmission dynamics of SGVs is essential for managing the risk of disease outbreaks. Australia offers an interesting ecological setting to examine SGV transmission as multiple viruses, including Akabane, Aino, Douglas (*O. schmallenbergense*), Peaton, and Tinaroo (*O. akabaneense*), are endemic.

Of the recognised vectors of SGVs in Australia, *Culicoides brevitarsis* is considered the most important due to its broad distribution, relatively high abundance, and a lifecycle closely associated with cattle [8–11]. While *C. brevitarsis* may persist year‐round in some regions, SGV transmission requires at least a month of sustained warm, humid conditions to support midge activity, which may cease during winter in regions where average minimum temperatures remain at or below 13.5°C [9, 11–13]. These meteorological constraints on vector ecology help explain regional differences in SGV transmission across northern and eastern Australia [14]. Tropical conditions in Queensland and the Northern Territory result in year‐round SGV transmission [4, 15]. In contrast, in the temperate state of New South Wales (NSW), SGV transmission occurs from November to May and ceases in winter (June to August) [11, 16]. In addition to this defined seasonal transmission period, all SGVs known to occur in Australia have also been detected in NSW.

Historical outbreaks in NSW demonstrate how the intermittent transmission of Akabane virus facilitates the maintenance of a susceptible population of naïve herds, leading to disease outbreaks when pregnant ruminants are infected. The earliest outbreaks of calves with arthrogryposis and hydranencephaly in NSW were reported in 1937 and 1956 [17, 18], predating the isolation of Akabane virus and its confirmed association with this syndrome decades later [19]. From the 1970s, cases were reported every 2 to 3 years, with outbreaks occurring every 8 to 10 years [4, 19–21]. The frequency of outbreaks of disease due to Akabane virus in NSW appears to have declined to approximately every 15–20 years, although cases continue to be reported in 2000, 2003, 2017, and most recently in 2024 [22–25]. In the absence of a commercially available vaccine, the intermittent transmission of the Akabane virus reinforces the importance of ongoing surveillance to identify at‐risk regions for outbreaks of congenital disease.

Surveillance for SGV transmission is essential for identifying at‐risk regions and livestock populations. Serological studies using sentinel herds, insect trapping, and passive disease reporting provide a comprehensive understanding of Akabane virus transmission across Australia [4]. The National Arbovirus Monitoring Program (NAMP) coordinates surveillance for veterinary‐important arboviruses [4, 26]. The detection of a Shamonda‐like virus in surveillance samples collected from a NSW sentinel cattle herd in 2017 also underscores the need for a clearer understanding of the year‐to‐year transmission patterns of SGVs and the specific distribution of each virus within this serogroup in NSW [7]. This study expands on the surveillance efforts from the NAMP by mapping the spatial and temporal distribution of Akabane, Aino, Douglas, Peaton, Tinaroo, and a Shamonda‐like virus using NSW sentinel herd data from 2005 to 2023. This is the first virus‐specific study to examine the long‐term distribution of each of the viruses within the Simbu serogroup, providing contemporary insights into SGV ecology and transmission dynamics in south‐eastern Australia.

## 2. Materials and Methods

### 2.1. NSW Sentinel Herds and Sample Collection

To investigate the spatial and temporal patterns of SGV transmission in NSW, serological data were obtained for samples collected for the NAMP and tested at the Virology Laboratory at the Elizabeth Macarthur Agriculture Institute. From 2000 to 2023, the NAMP enrolled ~30–40 sentinel cattle herds across NSW for each ‘monitoring period’, a 10‐month period from October to July of the following year, which coincides with known seasonal vector presence. Each sentinel herd should consist of at least 10 cattle born on the property, approximately 6 months of age at the time of first testing, and seronegative to bluetongue virus (BTV) and SGVs at the initial October sampling [4, 26]. For the NAMP, herd locations were selected to define the BTV transmission zone, with the frequency of blood collection ranging from monthly to quarterly, aligned with the anticipated BTV transmission by location [26].

Clotted blood was collected from each animal within the sentinel herd. After each collection, clotted blood samples were transported to the laboratory. The serum was separated by centrifugation (3000 g for 5 min at 4°C), stored at 4°C for testing, and archived at −20°C. Blood collection for each monitoring period spanned two calendar years (i.e. from October to July). However, for clarity, each monitoring period was referred to by the calendar year in which the final sample collection occurred (2000–2023).

**Figure 1:**
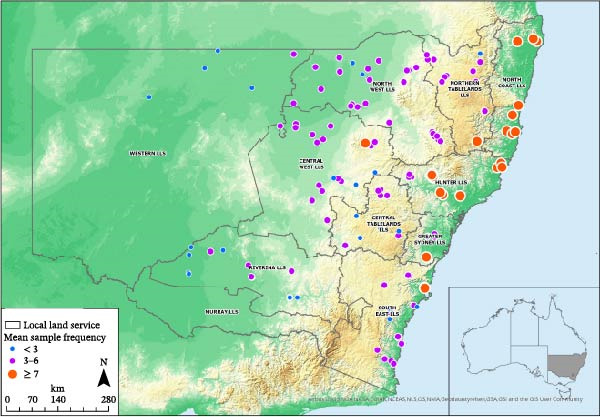
Sentinel herd locations and sampling frequency across New South Wales, 2000–2023. This map depicts all locations in New South Wales (NSW) where sentinel herds were enrolled during at least one of the 24 arbovirus monitoring periods from 2000 to 2023. Topography is shown using a gradient from green (low elevation) to yellow and brown (higher elevation), highlighting geographic features such as the Great Dividing Range (GDR). Point size and colour indicate the mean blood collection frequency at each location during the monitoring periods where a herd was enrolled. While a median of 37 herds was enrolled in each monitoring period (range: 27–43), not all locations were enrolled in every monitoring period. This map illustrates the complete set of herd locations that were sampled at any point throughout the study and does not reflect a single monitoring period.

For each herd, the final dataset included a herd identifier number, blood collection dates, and location details (recorded as place name, latitude, longitude, and region). The Local Land Service (LLS) regions were used as the regional descriptors to group locations with similar geoclimatic conditions across NSW (Figure 1): Central Tablelands, Central West, Greater Sydney, Hunter, Murray, North Coast, Northern Tablelands, North West, Riverina, South East, and Western [27]. To demonstrate SGV transmission at the herd location, a Simbu group–reactive antibody ELISA was used to monitor seroconversion. Results from the ELISA were recorded as follows: (1) number at risk (defined by the count of seronegative animals at the previous collection); (2) number of new seroconversions (animals that tested positive for the first time); and (3) the total number of animals that had seroconverted. To identify the transmission of specific SGVs, virus neutralisation tests (VNTs) were completed on herds where seroconversion was detected. The total count of positive antibody detections in the herd for each SGV in each VNT was recorded.

### 2.2. Serological Tests for SGVs

#### 2.2.1. Simbu Group–Reactive Antibody ELISA

Serum samples from each collection were tested using a Simbu group–reactive antibody ELISA, which was expected to detect antibodies to all SGVs because the amino acid sequence homology among nucleoproteins of viruses from the same serogroup is high [28–31]. From 2000 to 2016, an in‐house blocking ELISA for antibodies to SGVs was used. The method, adapted from Blacksell et al. [32], used cell culture–propagated Akabane virus as the antigen and a pool of mouse‐derived monoclonal antibodies that react with nucleoprotein epitopes that are common to viruses in the serogroup [33, 34]. As a blocking ELISA, the percentage inhibition (PI) values were calculated as the proportionate difference in the reactivity of the sample compared to the negative control. Samples with a PI value of ≥45% were considered positive for SGV antibodies, 35%–44% inconclusive, and <35% negative.

Following the emergence of the Schmallenberg virus in Germany in late 2011, the commercial Schmallenberg Virus Antibody Test Kit (IDEXX, Hoofddorp, Netherlands) was developed [35, 36]. This assay is capable of detecting antibodies for all known viruses in the Simbu serogroup, and this indirect ELISA was validated by testing animals infected with SGVs in Australia [37, 38]. From 2017, this commercial kit ELISA was used to test samples from the NSW sentinel herds and was performed according to the manufacturer’s instructions.

#### 2.2.2. VNTs

If seroconversion was detected in any animal using the Simbu group ELISA, then all samples from that sentinel herd collected during the final blood collection event for the monitoring period were tested using VNTs to detect antibodies for the five SGVs known to be present in Australia: Aino, Akabane, Douglas, Peaton, and Tinaroo (Table 1). From 2019, testing also included a Shamonda‐like virus found in NSW. The virus‐specific VNT results from 18 monitoring periods (2005–2007 and 2009–2023) were available for analysis.

**Table 1:** Details of the six Simbu serogroup orthobunyaviruses known to be present in Australia.

Virus species	Virus names	Test isolate	GenBank accessions^1^
*Orthobunyavirus ainoense*	Aino virus	B7974	MH734979, MH735036, MH735093
*Orthobunyavirus akabaneense*	Akabane virus	R7949	MH734959, MH735016, MH735073
	Tinaroo virus	CS153	MH734996, MH735053, MH735110
*Orthobunyavirus schmallenbergense*	Shamonda virus	L576	−
	Douglas virus	CS400	MH734987, MH735044, MH735101
*Orthobunyavirus peachesterense*	Peaton virus	CS347	MH734994, MH735051, MH735108

*Note:* The listed isolates were used in virus neutralisation tests to map the distribution of antibodies against each virus in New South Wales. ‘−’: Sequence data are not available on GenBank.

^1^All genome segment sequences for the Australian isolates were reported in Wang et al. [14].

VNT procedures followed the microtitre test method described by Della‐Porta et al. [39] with a modification of the cell culture from Vero to HmLu‐1 cells [40]. In brief, 1.8–2.5 log_10_ TCID_50_/50 µL of each virus preparation was combined with the serum samples and incubated at 37°C for 1 h. Following neutralisation, a suspension of HmLu‐1 cells was added and incubated at 37°C with 5% CO_2_ for 5 days. Plates were then examined for the presence or absence of cytopathology (CPE).

An initial screening test was performed using a 1/20 dilution of serum samples tested in triplicate. For samples in which neutralisation was detected at the screening dilution, the sample was titrated using a two‐fold dilution series ranging from 1/10 to 1/1280 to determine the titre. The titre of a sample was determined as the reciprocal of the highest dilution at which neutralisation was observed, as shown by the absence of CPE in at least one of two replicate wells. Further testing to determine titres that exceeded a dilution of 1/1280 was not conducted. The results for VNTs using different viruses for the same sample were interpreted independently, with the antibody titre reported against each SGV. The result for each VNT was considered positive when the titres were greater than 20, inconclusive when the titres were 10 or 20, and negative when neutralisation was not detected.

### 2.3. Mapping the Spatial Trends and the Distribution of SGVs Using the ELISA and VNT Results

The month of first recorded seroconversion was used to assess the seasonality of SGV transmission, using Simbu group–reactive ELISA results from 2000 to 2023. For each sentinel herd, the proportion of animals with antibodies detected in the ELISA was calculated as the total number of positive results divided by the number of animals at risk in the herd. This proportion, representing the seroprevalence in this herd, was plotted across NSW with a colour gradient indicating the month of seroconversion in R (v4.4.0) [41].

A distribution map for SGVs across NSW was created for each monitoring period using the ELISA and virus‐specific VNT results from 2005 to 2023 in R. The proportion of animals with antibodies detected in both ELISA and VNTs was calculated for each herd and used for spatial interpolation to estimate the distribution of SGVs across NSW. R packages ‘sp’ [42], ‘sf’ [43], and ‘terra’ [44] were used for spatial data handling and ‘gstat’ [45] for geostatistical methods.

To depict the distribution of SGV across NSW from the ELISA and VNT results, terrain features were included for spatial interpolation [46]. The elevation, slope, and aspect were matched into the dataset using the herd’s latitude and longitude in a 10 km × 10 km grid resolution. The sentinel herd’s latitude and longitude were used as the geometric centre to weight the spatial interpolation. To select the optimal model for spatial interpolation, the geostatistical methods (i.e. nearest neighbour, moving average, and Kriging) were compared based on their root mean square error (RMSE) values. Initially, for model selection, this assessment was restricted to data from the 2016 monitoring period, which had a high proportion of ELISA seroconversions, with antibodies detected only for Douglas and Peaton viruses in the VNT. Kriging using a spherical variogram provided the lowest RMSE values and was the best fit for spatial interpolation compared to the other models [47]. The Kriging method was used for every monitoring period to map the distribution of ELISA and VNT results across NSW. The RMSE values for spatial interpolation are provided in Table S1. The model results were converted into a raster format and masked to the NSW boundary to produce a choropleth map for each monitoring period of this dataset.

## 3. Results

Between 2000 and 2023, 46,489 samples were collected from 162 unique locations across NSW. Over the 24 monitoring periods, blood collection frequency varied by location (Figure 1), with samples collected from sentinel herds in 10 of the 11 NSW LLS regions. Along coastal NSW (North Coast, Hunter, and Greater Sydney), most herds were sampled in October and then monthly from December to July. In the adjacent inland regions (Northern Tablelands, North West, Central Tablelands, and Central West LLS), blood was collected every three to 4 months, typically in October, January, April, and June. In the Western and Riverina LLS regions, herds were generally sampled in October or December and June, with a third sampling occasionally in April. No herds were recruited from Murray LLS, the central southern area of NSW. A median of 37 herds were enrolled in each monitoring period (min. 27, Q1 34, mean 36.8, Q3 40, and max. 43). During each monitoring period, the median number of samples collected per herd at each blood collection event was 10 (min. 2, Q1 10, mean 11, Q3 10, and max. 35).

### 3.1. Transmission Patterns of SGVs Across NSW (2000–2023)

**Figure 2:**
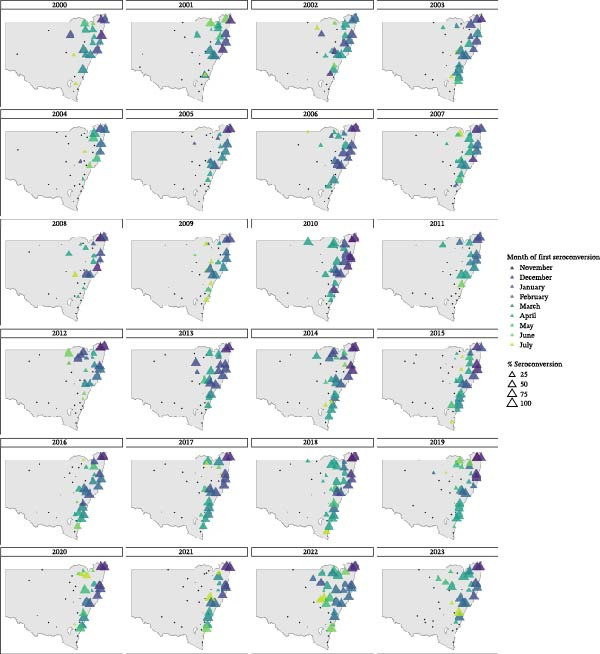
Simbu group virus (SGV) transmission across New South Wales (NSW) from 2000 to 2023. Each map represents the herd location where SGV seroconversion was detected by the serogroup‐reactive antibody ELISA in each monitoring period (October to July). Locations where no seroconversion occurred are shown as small black diamonds to illustrate the extent of sentinel herd monitoring completed in each period. The colour gradient indicates the month of the first seroconversion event (dark to light), and the size of the triangles represents the end of the monitoring period seroprevalence in the herd for each location.

SGV transmission was detected during every monitoring period from 2000 to 2023, as indicated by seroconversion using the Simbu group–reactive ELISA (Figure 2). As a general trend, in the early months of the monitoring period (November to February), seroconversions were detected in the coastal areas of the North Coast and Hunter LLS regions. In the latter months of the monitoring period (May to July), seroconversions were detected further south in other coastal areas of Greater Sydney LLS regions and further west in the neighbouring tablelands regions (Northern and Central Tableland LLS regions).

**Figure 3:**
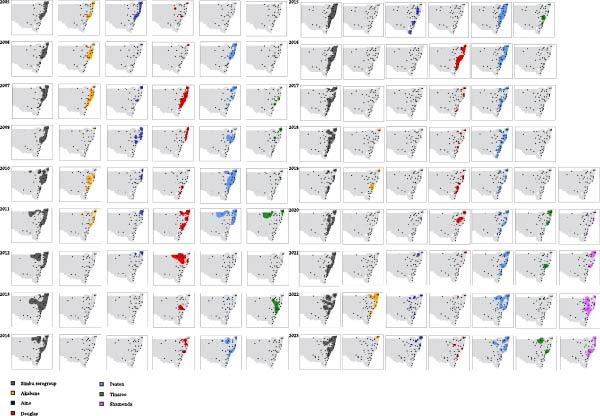
Distribution of Simbu group viruses in New South Wales based on sentinel herd testing with Simbu group–reactive ELISA and virus‐specific neutralisation tests (VNTs) over 18 monitoring periods (2005−2007 and 2009−2023). Black diamonds indicate sentinel herd locations to depict the extent of the monitoring program. Dark grey areas show transmission regions for Simbu group viruses from seroconversions detected by the group‐reactive ELISA. The distribution of specific viruses, Akabane, Aino, Douglas, Peaton, Tinaroo, and Shamonda, based on VNT results, is mapped in yellow, navy, red, light blue, green, and pink shading.

Over the 24 monitoring periods, SGV transmission was consistently detected in the coastal areas of NSW (North Coast, Hunter, and Greater Sydney LLS regions) (Figures 2 and 3). Transmission was occasionally detected in the Northern Tableland LLS region, especially during 2013, 2022, and 2023, and extended to the adjoining areas of the North West LLS regions. Elsewhere in NSW, transmission was inconsistent or absent. Transmission along the far south coast of NSW was occasionally observed between 2000 and 2014 but became more frequent in the South East LLS region from 2014 onwards. SGV transmission in the North West and Central West LLS regions was also sporadic and did not consistently coincide with southern extensions, especially between 2000–2003 and 2005–2008. No seroconversions were detected in herds from the western and central southwestern areas of NSW in the Western and Riverina LLS regions.

Where transmission was observed, the seroprevalence within sentinel herds ranged from 3.3% to 100%. At the end of the monitoring period, descriptive regional differences were apparent; herds in the North Coast LLS region generally showed higher within‐herd ELISA–positive proportions (range 85.7–100, along with herds in the adjacent Northern Tablelands LLS region (range 5.0%–87.5%). In contrast, herds in the North West and Central West LLS regions (range 0%–63.6%) and in the South East LLS region (range 0%–51.9%) had more variable and often lower observed seroprevalence.

### 3.2. Virus‐Specific VNTs in NSW Sentinel Herds (2005–2023)

The final blood samples collected from sentinel herds, where at least one animal seroconverted in the Simbu group–reactive ELISA during monitoring periods spanning 2005–2007 and 2009–2023, were tested using virus‐specific VNTs. Over these 18 monitoring periods, virus‐specific testing was completed for 78 unique locations across NSW. Following each 10‐month monitoring period, a median of 21 herds were tested (min. 10, Q1 18, mean 20.4, Q3 23.5, and max. 29). For each monitoring period, the final blood samples from all animals within a herd were tested, with a median of 11 samples per herd tested in the VNTs (min. 1, Q1 10, mean 11.5, Q3 12, and max. 27).

**Figure 4:**
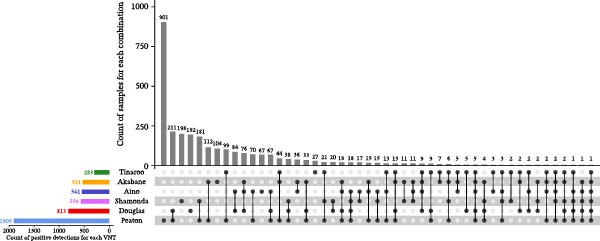
An UpSet plot illustrating the 49 unique combinations of virus neutralisation test (VNT) results and the frequency with which each combination was observed in 2785 VNT antibody‐positive serum samples collected over 17 transmission periods (2005–2007 and 2009–2023). The upper bar chart displays the count of samples for each observed combination, while the side bar chart shows the total number of positive detections per VNT. Counts have been annotated directly on the bars for clarity.

Of the 4458 serum samples tested, 62.5% were positive for antibodies in at least one VNT, with 49 combinations observed (Figure 4). Most samples were positive in only one VNT, with Peaton virus antibodies detected most frequently (901 samples). Antibodies were detected in one VNT for 1489 samples, in two VNTs for 892, in three for 319, in four for 74, and in five for 10 samples. Antibodies to all six viruses were detected in one animal.

### 3.3. Distribution of Each SGV Across NSW (2005–2023)

#### 3.3.1. Akabane Virus

Evidence of Akabane virus transmission was primarily restricted to coastal regions (North Coast, Hunter, and Greater Sydney LLS regions) between 2005 and 2007, overlapping with the distribution of Aino, Douglas, and Peaton viruses (Figure 3). Following 2007, transmission became increasingly sporadic. No detections were recorded between 2014 and 2016, and virus‐specific antibodies have only been intermittently detected since. In 2022, transmission extended to the Northern Tableland LLS region, but this pattern did not reoccur in 2023.

#### 3.3.2. Aino Virus

Aino virus exposure was initially limited to the coastal regions between 2005 and 2012, with detections often occurring in the same locations as Akabane, Douglas, and Peaton viruses (Figure 3). In more recent monitoring periods (2020–2023), evidence of transmission was more widespread across NSW. Antibodies against the Aino virus were detected in coastal regions reaching to the South East LLS, as well as inland extensions to the Northern Tablelands, neighbouring sentinel herds in the North West and Central West LLS areas.

#### 3.3.3. Douglas Virus

Douglas virus transmission was widespread across the SGV‐endemic regions of NSW. Antibodies were detected in coastal herds (North Coast, Hunter, Greater Sydney, and South East LLS) with inland extensions to Northern Tablelands, neighbouring sentinel herds in the North West and Central West LLS areas. The distribution of Douglas virus frequently overlapped with Peaton virus (Figure 3).

#### 3.3.4. Peaton Virus

Peaton virus transmission was detected in all 18 monitoring periods from 2005 to 2023 (excluding 2008) and had the widest distribution among the SGVs tested. This distribution consistently overlapped with that of Akabane, Aino, Douglas, Tinaroo, and Shamonda‐like viruses (Figure 3).

#### 3.3.5. Tinaroo Virus

Tinaroo virus exposure was detected sporadically, particularly in the same regions where the Peaton virus was present (Figure 3).

#### 3.3.6. Shamonda‐Like Virus

Since VNT testing for the newly recognised SGV started in 2019, Shamonda‐like virus exposure was initially restricted to herds in coastal areas of the North Coast, Hunter, and Greater Sydney LLS regions in 2019 and 2020. From 2021, the distribution of this Shamonda‐like virus expanded into the Northern Tablelands and neighbouring herds in the North West and Central West LLS regions (Figure 3). From 2022–2023, Shamonda‐like virus transmission not only overlapped with Peaton virus but extended further inland into the North West and Central West LLS regions and southward along the NSW coastline to the South East LLS region. Differences in the ELISA and VNT distributions for other SGVs suggest that the Shamonda‐like virus may have circulated in NSW from at least 2012.

## 4. Discussion

This serological study offers a contemporary spatiotemporal analysis of SGV transmission dynamics using over 46,000 samples collected from sentinel cattle across NSW over a 24‐year period. SGV transmission was detected in every monitoring period (October to July) between 2000 and 2023. Multiple SGVs cocirculated with transmission progressing from north to south in the coastal areas of the North Coast, Hunter, and Greater Sydney LLS regions. SGV transmission also often extended to the adjacent Northern Tablelands region. Over the past decade, an expansion of SGV transmission along the NSW coastline was observed with consistent antibody detection and increasing seroprevalence in the southern coastal areas of the South East LLS region. The Peaton virus was the most widespread and frequently detected SGV, while the recently recognised Shamonda‐like virus also had a widespread distribution since testing for its presence began in 2019. These findings underscore the dynamic nature of SGV transmission across NSW, where multiple viruses cocirculate within a window of seasonal vector activity during favourable conditions.

SGV transmission is closely linked to the ecology of *Culicoides* spp. biting midges. Early in each monitoring period, virus transmission was consistently detected in the North Coast LLS region from November to February (Figure 2), coinciding with the onset of optimal vector conditions in locations where overwintering populations of *C. brevitarsis* might be expected [12]. While occasional westward SGV transmission was observed beyond the coastal endemic zone (North Coast, Hunter, and Greater Sydney LLS regions), a consistent separation remains between these coastal regions and inland areas of central NSW (North West and Central West LLS regions) (Figure 3). Herds in elevated areas of the Northern and Central Tableland LLS regions experience little to no transmission (Figures 2 and 3), consistent with the altitude limits of vector dispersal [11].

As a system of mountains, plateaus, and valleys that separates the coastal from the central regions, the Great Dividing Range (GDR) presents a major barrier to SGV transmission [11, 48]. Wind‐assisted dispersal can carry midges across the GDR, particularly in areas where altitude decreases, such as in the Hunter Valley [10, 13, 49]. This westerly extension of SGV transmission to herds in the Central West LLS regions was observed in 2013, 2020, and 2022 (Figure 3). However, the distribution patterns in 2012 (Figures 2 and 3) also suggest that inland infections in the North West LLS region may result from SGV transmission moving down the western slopes of the mountain range from Queensland. Understanding these transmission dynamics is crucial for predicting and managing outbreaks of pathogenic SGVs, especially with changing meteorological conditions.

In contrast to the variable SGV transmission observed in North West and Central West LLS regions, the far south coast of NSW (South East LLS region) has shown an emerging pattern of consistent seasonal seroconversion. Since 2014, SGV transmission has repeatedly extended southward along the NSW coastline, with increasing seroprevalence in the South East LLS region (Figure 2). This trend may reflect the influence of increasingly mild winters, which are likely extending the duration of favourable temperature conditions for insect vectors on the far south coast of NSW, possibly by several weeks each year, compared to historical patterns [50–52]. Historically, intermittent Akabane virus transmission was observed on the south coast of NSW during the 1980s and 1990s, with associated outbreaks of congenital disease [8–10, 12, 19].

These changing patterns of SGV transmission also have important implications for livestock immunity and disease risk. The absence of Akabane virus seroconversions between 2014 and 2016 (Figure 3), followed by cases of congenital malformations in 2017 [24], illustrates how sporadic virus distribution can leave herds vulnerable. Without recent and regular exposure, a large proportion of animals may remain immunologically naïve, increasing the likelihood of congenital disease when Akabane virus transmission resumes during critical stages of gestation [10, 13, 53]. In the absence of vaccination, consistent exposure to Akabane virus would reduce foetal disease risk by enabling animals to develop immunity before breeding. However, the distinct distribution patterns of each SGV across NSW could result in varying levels of exposure (Figure 3). These findings highlight the importance of long‐term, virus‐specific surveillance to identify potential gaps in the immunity to pathogenic viruses and predict periods of increased disease risk.

Despite the consistent pattern of seasonal SGV transmission across NSW, seasonal variation in the transmission of specific viruses was observed. Antibodies to more than one virus were found in 46.5% of VNT‐positive samples, suggesting sequential or possible coinfections within a single monitoring period (Figure 4). Similar findings have been reported in Africa [54, 55], and longitudinal serology in NSW has demonstrated that cattle can be sequentially infected with three or more SGVs within a 6–10 week window [56]. Overall, antibodies to Peaton virus were the most frequently detected compared to the other SGVs (Figure 4). This contrasts with earlier studies in NSW, where the Peaton virus transmission was observed intermittently and at low seroprevalence [56]. These shifts in the transmission of specific SGVs raise questions about the mechanisms of virus dominance in south‐eastern Australia.

Identifying the dominant SGV and understanding the competition between the viruses in the same ecological niche is crucial for disease risk management, particularly because multiple SGVs of differing pathogenicity cocirculate in NSW. The decline in Douglas virus antibody detections appears to coincide with the increasing seroprevalence of the Shamonda‐like virus (Figure 3). Douglas virus and Shamonda virus are classified as member viruses of the species complex *O. schmallenbergense* [57]. In vitro studies suggest that selective pressures during transmission between insect and mammalian hosts could favour antigenic variants within virus species to facilitate the evasion of existing neutralising antibodies [58]. These interactions could allow one virus to displace another, leading to the dominance of less immunologically familiar SGVs [59]. In addition to this viral competition, the cocirculation of multiple SGVs facilitates genetic reassortment.

Genetic reassortment among endemic SGVs in NSW is possible within an ecological niche where multiple viral species circulate. Reassortant viruses have been reported for Akabane and Tinaroo viruses [34, 60, 61], and the simultaneous infection of midges or the mammalian hosts allows RNA segments to be exchanged during replication, potentially producing novel variants with altered virulence or pathogenicity [58, 59]. Detection and characterisation of such reassortment events would require genome‐level analysis of circulating SGVs, which was beyond the scope of this serological study. However, the observed cocirculation and shifting seroprevalence patterns (Figure 3) underscore the importance of future molecular investigations to contextualise these serological findings, particularly in establishing whether a variant virus has arisen from a local reassortment event or from a long‐distance introduction from Northern Australia or Southeast Asia.

The potential for exotic SGV incursions into Australia is supported by both the long‐distance dispersal of *Culicoides* midges during extreme weather events and genetic similarities between Australian and Asian SGVs. *C. brevitarsis* can be carried by wind for more than 100 km across water and survive for up to 36 h, creating a viable pathway for the introduction of SGVs from Southeast Asia into northern Australia [16, 62–64]. Genomic evidence supports this, with Aino and Akabane virus strains in Australia closely resembling Japanese isolates, suggesting past introductions or origin from a common source [34, 61]. Therefore, the detection of a Shamonda‐like virus in NSW highlights the need for genomic characterisation and molecular epidemiology, including phylogenetic analysis, to determine the origins of this virus and assess the probability of exotic introduction.

This study provides an update on the distribution of SGVs in NSW, but several methodological constraints limit the interpretation of transmission timing and virus interactions within the monitoring period. Sampling occurred every 3–4 months for sentinel herds in the Northern Tablelands, North West, Central West, and South East LLS regions (Figure 1), which limited the precision in identifying the onset of transmission (Figure 2). VN testing was completed on only the final sample collected in the monitoring period, which limits any interpretation for cross‐reactivity, coinfection, or sequential infection when antibodies to multiple SGVs were detected (Figure 3). Previous longitudinal studies in NSW employed more frequent sampling and virus isolation to define the timing of infection for each SGV [56]. Finally, the extensive serum collection used in this study represents a valuable resource for future virological investigations. Targeted PCR‐based assays and/or unbiased high‐throughput sequencing of samples collected immediately prior to, or during, the earliest seroconversion events would likely enable direct identification of the viral species responsible for the observed antibody responses.

Our study did not include analysis of entomological data (i.e. *C. brevitarsis* abundance) or meteorological variables (i.e. minimum daily temperatures and evapotranspiration), which would be necessary to produce predictive models to estimate the disease risk across NSW. As a vector‐borne pathogen, disease risk is facilitated by these factors; for instance, drought conditions lead to suppressed virus transmission and reduced herd immunity, which subsequently facilitate the transmission of SGVs [65] when more favourable weather patterns resume. Drought conditions also increase the disease risk due to the movement of susceptible animals [66]. While the maps in this study do not represent disease risk but rather virus distribution, this long‐term dataset offers a foundation for using virus‐specific surveillance to identify immunity gaps and anticipate periods of increased disease risk in NSW.

## 5. Conclusion

This 24‐year surveillance study reveals that SGV transmission occurs annually during the 10‐month monitoring period (October to July), with a clear seasonal progression from north to south along the NSW coastline. Transmission in the Northern Tablelands and neighbouring herds in the North West LLS regions was sporadic, increasing the risk of congenital disease when naïve pregnant animals are exposed. In contrast, the consistent detection of SGVs along the far south coast since 2014 suggests an expansion of the coastal distribution of these viruses, potentially influenced by warming meteorological conditions. Over the study period, Peaton virus emerged as the most widespread SGV, while the Douglas virus declined in prevalence. A Shamonda‐like virus was widely distributed since testing started for this SGV in 2019. These findings highlight the need for virus‐specific surveillance to identify the dominant SGV in the transmission and to detect emerging variants or exotic incursions.

## Author Contributions

Conceptualisation: Tiffany W. O’Connor, Paul M. Hick, Jenny‐Ann L. M. L. Toribio, Deborah S. Finlaison, and Peter D. Kirkland. Methodology: Tiffany W. O’Connor, Paul M. Hick, Jenny‐Ann L. M. L. Toribio, Deborah S. Finlaison, and Peter D. Kirkland. Software: Tiffany W. O’Connor. Validation: Tiffany W. O’Connor, Paul M. Hick, Jenny‐Ann L. M. L. Toribio, Deborah S. Finlaison, and Peter D. Kirkland. Formal analysis: Tiffany W. O’Connor, Paul M. Hick, Jenny‐Ann L. M. L. Toribio, Alison J. Peel, Deborah S. Finlaison, and Peter D. Kirkland. Investigation: Tiffany W. O’Connor, Paul M. Hick, Jenny‐Ann L. M. L. Toribio, Alison J. Peel, Deborah S. Finlaison, and Peter D. Kirkland. Resources: Deborah S. Finlaison and Peter D. Kirkland. Data curation: Deborah S. Finlaison and Peter D. Kirkland. Writing – original draft preparation: Tiffany W. O’Connor, Paul M. Hick, Jenny‐Ann L. M. L. Toribio, Deborah S. Finlaison, and Peter D. Kirkland. Writing – review and editing: Tiffany W. O’Connor, Paul M. Hick, Jenny‐Ann L. M. L. Toribio, Alison J. Peel, Deborah S. Finlaison, and Peter D. Kirkland. Visualisation: Tiffany W. O’Connor, Paul M. Hick, Jenny‐Ann L. M. L. Toribio, Alison J. Peel, Deborah S. Finlaison, and Peter D. Kirkland. Supervision: Paul M. Hick, Jenny‐Ann L. M. L. Toribio, Alison J. Peel, Deborah S. Finlaison, and Peter D. Kirkland. Project administration: Paul M. Hick, Jenny‐Ann L. M. L. Toribio, Deborah S. Finlaison, and Peter D. Kirkland. Funding acquisition: Deborah S. Finlaison and Peter D. Kirkland.

## Funding

This study was funded by the New South Wales Department of Primary Industries (NSW DPI). Alison Peel J. was supported by a Sydney Horizon Fellowship. Open access publishing facilitated by New South Wales Department of Planning Housing and Infrastructure, as part of the Wiley ‐ New South Wales Department of Planning Housing and Infrastructure agreement via the Council of Australasian University Librarians.

## Ethics Statement

Ethical approval was not required for this study as no human or animal subjects were directly involved.

## Conflicts of Interest

The authors declare no conflicts of interest.

## Supporting Information

Additional supporting information can be found online in the Supporting Information section.

## Supporting information


**Supporting Information** Table S1: The root mean square error values for the spherical variograms used in the spatial interpolation to delineate the annual spatial distribution of Simbu group viruses in New South Wales.

## Data Availability

The data that support the findings of this study are available from Animal Health Australia. Restrictions apply to the availability of these data, which were used under license for this study. Data are available from https://animalhealthaustralia.com.au/national-arbovirus‐monitoring‐program/with the permission of Animal Health Australia.
