# Supplementary material for: The Dynamic Transmission of Simbu Group Viruses in New South Wales, Australia
Source: Transbound Emerg Dis. 2026 Jun 24;2026:9214955. doi: 10.1155/tbed/9214955 (PMC13292022; doi:10.1155/tbed/9214955)
Supplement: Supplementary file 1 — Supporting Information Table S1: The root mean square error values for the spherical variograms used in the spatial interpolation to delineate the annual spatial distribution of Simbu group viruses in New South Wales. [file TBED-2026-9214955-s001.docx]

# Supplementary materials

**Supplementary 1.** The root mean square error values for the spherical variograms used in the spatial interpolation to delineate the annual spatial distribution of Simbu group viruses in New South Wales.

|  | Assay | | | | | | |
| --- | --- | --- | --- | --- | --- | --- | --- |
| Year | Serogroup ELISA | Akabane VNT | Aino VNT | Douglas VNT | Peaton VNT | Tinaroo VNT | Shamonda VNT |
| 2005 | 2.09 | 2.84 | 1.90 | 2.50 | 0.17 | 0.25 | *NT* |
| 2006 | 4.17 | 1.92 | 0.25 | 1.34 | 2.66 | 0.18 | *NT* |
| 2007 | 3.78 | 2.42 | 0.22 | 1.76 | 0.50 | 0.39 | *NT* |
| 2008 | 1.86 | *NT* | *NT* | *NT* | *NT* | *NT* | *NT* |
| 2009 | 2.13 | 0.37 | 1.26 | 1.95 | 0.69 | 0.39 | *NT* |
| 2010 | 4.55 | 0.67 | 0.86 | 2.38 | 2.53 | 0.49 | *NT* |
| 2011 | 2.26 | 1.63 | 0.54 | 2.08 | 2.57 | 0.95 | *NT* |
| 2012 | 3.14 | 0.65 | 1.13 | 1.56 | 3.34 | 0.77 | *NT* |
| 2013 | 4.09 | 0.32 | 0.33 | 1.09 | 3.84 | 0.37 | *NT* |
| 2014 | 3.87 | *ND* | 0.17 | 1.33 | 4.31 | 0.32 | *NT* |
| 2015 | 2.47 | *ND* | 0.40 | 0.16 | 2.33 | 0.91 | *NT* |
| 2016 | 2.74 | *ND* | *ND* | 2.00 | 2.01 | *ND* | *NT* |
| 2017 | 4.55 | 1.02 | 0.50 | 1.49 | 3.10 | 0.41 | *NT* |
| 2018 | 4.49 | 1.37 | *ND* | 1.74 | 4.20 | 1.34 | *NT* |
| 2019 | 4.65 | 1.20 | 0.70 | 1.95 | 4.13 | 0.16 | 2.34 |
| 2020 | 4.11 | 0.11 | 0.17 | 0.96 | 3.18 | 0.20 | 3.81 |
| 2021 | 3.80 | *ND* | 0.17 | 0.28 | 4.55 | 0.13 | 2.85 |
| 2022 | 4.89 | 1.7 | 0.48 | 0.98 | 1.79 | 0.11 | 4.11 |
| 2023 | 3.47 | 0.40 | 1.87 | 1.25 | 2.65 | 0.24 | 1.48 |

NT – Not tested; ND – Not detected
